# Impact of anticancer chemotherapy on the extension of beta-lactamase spectrum: an example with KPC-type carbapenemase activity towards ceftazidime-avibactam

**DOI:** 10.1038/s41598-020-57505-w

**Published:** 2020-01-17

**Authors:** Claire Amaris Hobson, Stéphane Bonacorsi, Didier Hocquet, André Baruchel, Mony Fahd, Thomas Storme, Raksamy Tang, Catherine Doit, Olivier Tenaillon, André Birgy

**Affiliations:** 1 Université de Paris, IAME, INSERM, F-75018 Paris, France; 2 AP-HP, Hôpital Robert Debré, Service de Microbiologie, F-75019 Paris, France; 3 Hygiène Hospitalière, UMR CNRS 6249, Université de Bourgogne Franche-Comté, Besançon, France; 4 Service d’Immuno-Hématologie Pédiatrique, Hôpital Robert Debré, AP-HP, Paris, France; 5 Pharmacie Hospitalière, Hôpital Robert Debré, AP-HP, Paris, France

**Keywords:** Molecular evolution, Antimicrobial therapy, Chemotherapy

## Abstract

Through their action on DNA replication, anticancer chemotherapies could increase the basal mutation rate in bacteria and increase the risk of selecting antibiotic resistant mutants. We investigated the impact of several drugs on a beta-lactamase model using KPC-type carbapenemase-producing *Enterobacteriaceae*. We studied the impact of anticancer chemotherapies used in pediatric hematologic malignancies on 7 clinical isolates of *Enterobacteriaceae* producing KPC-type carbapenemases. We compared the mutation rates from cultures with/without chemotherapy on ceftazidime-avibactam, rifampicin and ceftazidime-avibactam combined with meropenem media. Mechanisms of ceftazidime-avibactam resistance were explored on a subset of mutants. After exposure to some cytotoxic molecules, the bacterial mutation rates leading to ceftazidime-avibactam and to rifampicin resistance increased up to 10^4^-fold while we observed no emergence of resistant mutants (frequency of <10^−10^) on a meropenem combined with ceftazidime-avibactam media. Compared to the parental strains, an increased susceptibility to meropenem was observed in the ceftazidime-avibactam resistant mutants. The *bla*_KPC_ genes of ceftazidime-avibactam mutants harbored either mutations, deletions or insertions, especially in the region encoding the Ω-loop of the KPC-type carbapenemase. Anticancer chemotherapy can increase the mutation rates of bacteria accelerating the extension of KPC-type carbapenemases towards ceftazidime-avibactam, one of the last resort antimicrobial chemotherapy.

## Introduction

Patients treated with anticancer chemotherapy drugs (ACD) are vulnerable to infectious diseases due to immunosuppression and to the direct impact of ACD on their intestinal microbiota^1,2^. Some anticancer drugs like mitomycin C are known to have a bacteriostatic or bactericidal effect^3–6^ and can also be mutagenic on bacteria^3^. It is suggested that the dysbiosis can participate to intestinal mucositis^7^ that enhances the risk of bacterial translocation to the blood, requiring an antibiotic therapy, which may in turn favor the emergence of antibiotic resistant bacteria. The growing carriage of Carbapenem-Resistant *Enterobacteriaceae* (CRE) increases the risk of invasive infections with these resistant bacteria responsible for a mortality rate over 30%^8^. *Klebsiella pneumoniae* carbapenemase (KPC) was described for the first time in 1996^9^. It is now a common carbapenem resistance mechanism among *Enterobacteriaceae* in the USA, Israel, Asia, Latin America, and South Europe, reaching a prevalence rate of 66.5% among *K. pneumoniae* isolated in Greece^10,11^. Carbapenem-resistant *K. pneumoniae* bacteremia are responsible for 73% of 30-day mortality in cancer patients^12^. The recent combination ceftazidime-avibactam (CZA) has been approved in 2015 in the USA and in 2016 in Europe^13,14^. CZA demonstrates excellent *in vitro* activity against CRE of KPC-type, and is associated with a decreased mortality rate in treated patient^8,15^. Its efficacy, broad antibacterial spectrum and safety, led to an increase in its use, notably for immunosuppressed patients treated with ACD^16^. Unfortunately, some mutations in *bla*_KPC_ or in genes encoding porins lead to resistance to CZA, thus limiting the benefit of this last resort strategy^13,17,18^. Moreover, several ACD interfere with DNA replication and increase the mutation rate of eukaryotic cells. It has been showed *in vitro* that anticancer chemotherapy drugs could enhance the emergence of antibiotic-resistant pathogenic bacteria, mostly through activation of the SOS response^2,19^. The alkylating agent dacarbazine, the topoisomerase inhibitors epirubicin and daunorubicin, and the pyrimidine analogue azacitidine were shown to accelerate the bacterial evolution^19^. We thus hypothesized that ACD treatment could accelerate the modification of beta-lactamase spectrum and thus the emergence of CZA-resistant mutant in KPC-producing *Enterobacteriaceae*.

In this study, we investigated the impact of several ACD on beta-lactamase evolution. We chose to evaluate the emergence of CZA-resistant mutants in KPC-producing *Enterobacteriaceae*. We subsequently evaluated an antimicrobial combination to limit the emergence of resistant mutants to this last resort antibiotic. Last, we sequenced and cloned the *bla*_KPC_ genes of the CZA-resistant mutants to identify the mutations accounting for the enhance resistance to CZA.

## Results

### Mutation rate

**Figure 1:**
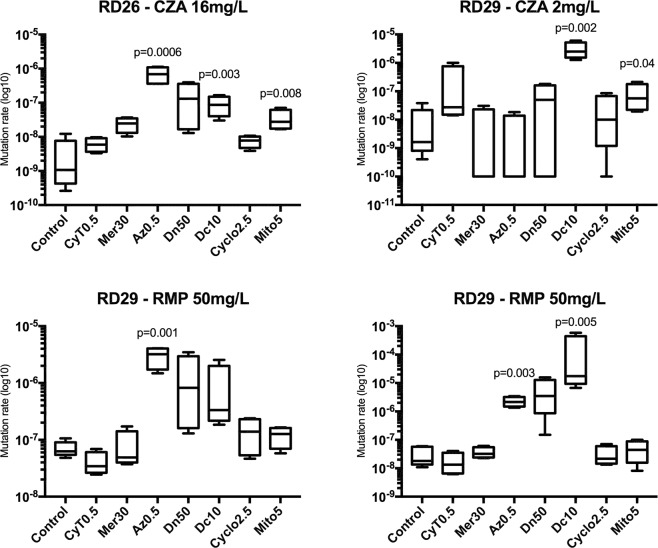
*In vitro* impact of anticancer drugs on the emergence of ceftazidime-avibactam (CZA) resistance in KPC-type carbapenemase producing Enterobacteriaceae. Cytarabine (CyT0.5), mercaptopurine (Mer30), azacitidine (Az0.5), daunorubicin (Dn50), dacarbazine (Dc10), cyclophosphamide (Cyclo2.5), and mitoxantrone (Mtx5) were used at the concentrations of 0.5, 30, 0.5, 50, 10, 2.5, and 5 mg/L respectively. Frequency of emergence of CZA- and rifampicin- (RMP-) mutants from *E. cloacae* KPC-3 (RD26) and *E. coli* KPC-2 (RD29) were determined on media containing antibiotics as follow: (**A**) RD26 on CZA 16 mg/L. (**B**) RD29 on CZA 2 mg/L. (**C**) RD26 on RMP 50 mg/L. (**D**) RD29 on RMP 50 mg/L. Ratio of means ± SD from four independent experiments. The *p*-value is indicated when differences compared to the control are significant (p < 0.05). Statistical analysis was performed using an MSS-MLE method followed by a *t*-student test.

The culture of *Enterobacter cloacae* KPC-3 (RD26) and *E. coli* KPC-2 (RD29) with ACD modified the frequency of resistant mutants to CZA and rifampicin in a variable manner depending on the isolates and the cytotoxic molecules. Resistance to rifampicin is a classical model used to evaluate mutation rates^20,21^. Control conditions without chemotherapy molecule showed that the strains have a normal mutational rate against rifampicin^20^. Compared to the control culture without ACD, the frequency of emergence of rifampicin-resistant mutants and of CZA-resistant mutants increased up to 10^4^ and to 10^3^ after exposure to some cytotoxic molecules (Fig. 1 and Supplementary Table S1).

In details, the frequency of emergence of CZA-resistant mutants from *E. cloacae* RD26 significantly increased after incubation with azacitidine (p = 0.0006), dacarbazine (p = 0.003), and mitoxantrone (p = 0.008). Azacitidine had the highest impact on mutation rates with an increase up to 10^3^-fold. Dacarbazine (p = 0.002) and mitoxantrone (p = 0.04) significantly increased the frequency of CZA-resistant mutants from *E. coli* KPC-2 (isolate RD29), up to 10^3^-fold increase for dacarbazine (Supplementary Table S2).

Dacarbazine increased the rate of rifampicin-resistant mutants (p = 0.005), with a maximum of 10^4^-fold increase (Supplementary Tables S1 and S2) for *E. coli* RD29. Azacitidine also had a significant impact for both strains (p = 0.001 for *E. cloacae* RD26, and p = 0.003 for *E. coli* RD29) (Supplementary Tables S1 and S2) while mitoxantrone, cyclophosphamide, daunorubicin, mercaptopurine and cytarabine had no significant effect.

Concerning meropenem, whatever the ACD used with the RD26 isolate, no mutation rate could be determined since confluent growth was observed at the concentration used. However, no resistant mutant was observed on the medium with CZA combined with meropenem and therefore the mutation rate is <10^−10^ (Supplementary Tables S1 and S2).

### Confirmation of the ACD impact on the mutation rate with azacitidine as an example, on 5 other strains

In order to evaluate the capability of ACD, and especially azacitidine which is one of the most used molecules in pediatric hematologic malignancies, to increase mutation rate of different species or isolates, five non-related clinical isolates of different sequence types of *K. pneumoniae* (Supplementary Tables S3 and S4) were tested against azacitidine 0.5 mg/L. Similar results with increased frequencies of rifampicin- and CZA-resistant mutants were observed but no resistant mutant on the medium combining meropenem and CZA.

### Confirmation of the ACD impact on the MIC increase by comparing parental isolates with transformants

The MICs of CZA on nine CZA-resistant mutants derivatives of RD26 selected randomly were 4- to 16-times higher than that of their parental isolate. This resulted in some MICs incompatible with therapeutic use. In contrast, the meropenem MIC decreased subsequently, from 16 mg/L with the parental isolate to 0.094–2 mg/L in the CZA-resistant mutants. The MIC of meropenem combined with CZA stayed very low (≤0.064 mg/L). MICs of CZA, meropenem, and meropenem/CZA performed on *E. coli* Top 10 carrying the *bla*_KPC_ genes borne in the plasmid pBR322 gave similar results. A similar level of *bla*_KPC_ mRNA transcription was observed for the recombinant mutants compared to their recombinant parental strain (Supplementary Table 5) suggesting similar KPC expression. Resistance to CZA was therefore very likely to be due to *bla*_KPC_ gene alteration (Table 1). To explore the *bla*_KPC_ gene mutation, we sequenced it in these nine RD26 CZA-resistant mutants as well as in seven transformants of *E. coli*. These revealed eithers point mutations, deletions, or insertions in the region of the omega-loop of the KPC (Table 1). Indeed, two mutants carried the mutation R164P, two mutants were mutated in position 179 (D179H and D179Y), and one mutant carried the mutation T243M. We also observed mutations leading to the insertion of 5 amino acids (SAIPG) between G175 and D176, of three amino acids (DDK) between K273 and Y274 and to the deletions of three amino acids (ARD) between D176 and T180 and of two amino acids (GV) between C238 and Y241 (Table 1).

**Table 1:** MICs (mg/L) of ceftazidime-avibactam (CZA), meropenem and meropenem in combination with CZA determined on *E. cloacae* KPC-3 (RD26), and a subset of CZA resistant mutants obtained after exposure to cytotoxic agents (Dacarbazine (Dc10), azacitidine (Az0.5), cyclophosphamide (Cyclo2.5), mitoxantrone (Mtx5), daunorubicin (Dn50) used at the concentrations of 10, 0.5, 2.5, 5 and 50 mg/L respectively), and on *E. coli* TOP10 electroporated with pBR322 plasmid harboring the same *bla*_KPC_ genes. MICs of antibiotics and mutations in *bla*_KPC_ observed in *E. cloacae* KPC-3 (RD26) of CZA-resistant mutants and transformants_a_, selected after exposure to cytotoxic agents.

Cytotoxic agent	Isolates	MIC (mg/L)			Identified mutation in *bla*_KPC_	Reference
		Ceftazidime-Avibactam	Meropenem	Meropenem + Ceftazidime-Avibactam		
—	RD26 (KPC-3)	4	16	0.023		
	Top10-pBR322-RD26	1	1.5	0.016		
Dc10	RD26-1	16	0.094	0.047	R164P	Winkler *et al*.
	—	—	—	—		
Dc10	RD26-2	32	0.094	0.032	R164P	Winkler *et al*.
	Top10-pBR322-RD26-2	24	0.032	0.012		
Az0.5	RD26-3	16	0.125	0.047	D179H	This study
	Top10-pBR322-RD26-3	24	0.032	0.016		
Dc10	RD26-4	32	0.125	0.064	D179Y	Livermore *et al*.
		—	—	—		
Dc10	RD26-5	12	0.38	0.023	T243M	Haidar *et al*.
	Top10-pBR322-RD26-5	12	0.064	0.016		
Mito5	RD26-6	32	2	0.032	G175-S-A-I-P-G-D176	This study
	Top10-pBR322-RD26-6	24	0.023	0.016		
Cyclo2.5	RD26-7	48	0.094	0.047	D176-∆A177-∆R178-∆D179-T180	This study
	Top10-pBR322-RD26-7	24	0.064	0.016		
Dc10	RD26-8	32	1	0.032	K273-D-D-K-Y274	KPC-29
	Top10-pBR322-RD26-8	24	0.125	0.023		
Dn50	RD26-9	32	0.125	0.047	C238-∆G239-∆V240-Y241	This study
	Top10-pBR322-RD26-9	64	0.023	0.023		

^a^Transformants were obtained by cloning *bla*_KPC_ (wild-type and mutated) in pBR322 plasmids and electroporated in *E. coli* Top10.

## Discussion

We showed here that certain ACD can stimulate the bacterial mutagenesis and can lead to mutations in *bla*_KPC_. Some of these mutations were previously shown to increase the catalytic efficiency of KPC towards ceftazidime resulting in the failure of ceftazidime-avibactam^22,23^.

Anticancer drugs can have genotoxic effects^24^ and thus stress the bacteria, through their impact on DNA and cell division. Stress activates the SOS response that increases the mutation rates of bacteria^19,21,25^. CZA may offer a significant advance over antimicrobials agents which may keep *in vitro* activity against CRE, such as colistin, fosfomycin, and tigecycline but which are limited by concerns over efficiency and/or toxicity^16^. Although CZA is considered as one of the last resort treatment in case of infection with KPC-producing strains^8^ its use may lead to the selection of resistant mutants in patients^15,17^. Reports on the emergence of CZA-resistant CRE soon after its launch led to the publication of a worrying report on rapid risk assessment in June 2018 by the European Centre for Disease prevention and Control^26^.

In this study, we observed that when bacteria are incubated with certain ACD such as azacitidine or dacarbazine, the increase frequency of resistant mutants (up to 10^−6^) and their high MIC to CZA are particularly worrying as it could lead to the treatment’s failure in case of severe infection.

As *E. cloacae* RD26 was not susceptible to meropenem, CZA would have been an option for the treatment in case of infection. Hence, we observed confluent growth on the medium containing meropenem at therapeutic concentration. A valuable result to point out is the absence of selection of cross-resistant mutants to the association CZA-meropenem. Indeed, whatever the mutation rate after a culture with ACD and selection on antibiotic medium, we found no emergence of resistance to this association at threshold detection of 10^−10^. This is of importance as it widens the therapeutic possibilities to reduce the risk of selection of resistant mutants. Avibactam protects meropenem from hydrolysis by the KPC ß-lactamase and restores the ceftazidime activity in the case of production of ESBL or AmpC ß-lactamases. Furthermore, a tradeoff between carbapenemase activity and resistance to CZA has already been described and was confirmed by our MIC results (Table 1) suggesting that the association meropenem with CZA is an optimal therapy to counteract the carbapenemase activity of the KPC ß-lactamase, and enables the meropenem and ceftazidime activity^15,22,23,27–29^. The association of meropenem and vaborbactam is promising but not yet available worldwide.

The sequencing of *bla*_KPC_ of CZA-resistant mutants identified point mutations, insertions, or deletions. Some of these have already been described after *in vivo* or *in vitro* selection^13,27,30^. These include mutations in position 164 and 179 which are located in the Ω-loop of this class A ß-lactamase. The Ω-loop (residues 164–179) is a hot spot for substitutions that extend the substrate spectrum of many class A enzymes. It contains Glu_166_ and Asn_170_, two essential residues required for priming a water molecule for deacylation of the ß-lactam. Mutations in the Ω-loop can modify salt bridge (in particular between Arg_164_ and Asp_179_) and thus enhances the flexibility of the loop allowing a better binding and hydrolysis of ceftazidime. Similarly, insertions or deletions that occur in the Ω-loop modify its structure and steric bulk^22,23,29^ and thus modify the access of ceftazidime. The effect of methionine for threonine substitution at position 243 is unclear^27^ as well as deletions of 2 amino acids (GV) between C238 and Y241. Due to the proximity with β3-strand (positions 234 to 242), these mutations could increase flexibility of the β3 strand and allow the aminothiazole ring of ceftazidime to sink deeper into the active site upon binding. Finally, the effect of the insertion of 3 amino-acid (DDK) between positions 273 and 274 is not known, but its spatial proximity to the active site is probably significant^31^.

Given the high mutation rates and potential clinical implications of therapeutic failure, it seems therefore reasonable to consider an association such as CZA with meropenem in the event of treatment of a severe infection by a KPC-producing *Enterobacteriaceae* in a patient treated with ACD and especially with dacarbazine, azacitidine, and mitoxantrone.

One of the biggest difficulties in carrying out this study was to choose the concentrations of chemotherapy molecules. Indeed, there are few data on therapeutic concentrations in pediatrics and to our knowledge none on concentrations obtained in stool. We therefore chose concentrations compatible with the blood concentrations^19,32,33^. We hypothesized that the diffusion of ACD in the intestinal compartment could increase the diversity of intestinal microbial population by modifying the basal mutation rate. Resistant mutants can then be selected after an antibiotic treatment, even not administered concomitantly with the ACD treatment. This remains to be evaluated *in vivo*.

In conclusion, using two KPC-producing *Enterobacteriaceae* strains as a model, we found that *in vitro* incubation with certain ACD (*i.e*. azacitidine, dacarbazine, mitoxantrone) enhance the bacterial mutagenesis and the subsequent emergence of antibiotic-resistant mutants. We confirmed the capability of azacitidine to generate high rates of mutants on five different sequence types of KPC-producing *K. pneumoniae*. Further studies including a larger number of bacterial isolates and cytotoxic molecules should be considered as well as *in vivo* assays.

Considering the rapid evolution of antibiotic resistance and the importance of beta-lactams in therapeutic arsenal, it seems essential to identify all factors that contribute to the evolution of antimicrobial resistance. Using KPC beta-lactamase, our study is an *in vitro* example that some ACD can increase mutational rate leading to CZA resistance. This observation challenges the therapeutic management of patients when dealing with KPC-type carbapenemase producing *Enterobacteriaceae*.

## Methods

### Bacterial isolates

In a first part, we studied two multiresistant *Enterobacteriaceae* producing KPC-type carbapenemases: a clinical isolate of *Enterobacter cloacae* harboring *bla*_KPC-3_ (RD26) and of *Escherichia coli* harboring *bla*_KPC-2_ (RD29). MICs determined by the E-test® method (bioMérieux, France) on Mueller Hinton agar (MHA) were of 16, and 0.25 mg/L for meropenem, and 4 and 0.5 mg/L for CZA for RD26 and RD29, respectively.

In a second part, we studied five multiresistant KPC-producing *K. pneumoniae* clinical isolates: RD27 (*bla*_KPC-3_), DD34 (*bla*_KPC-2_), DD36 (*bla*_KPC-2_), DD37 (*bla*_KPC-2_), and DD38 (*bla*_KPC-3_) whose MIC to meropenem were of >32, 6, 16, 6, and 1 mg/L and to CZA of 8, 4, 2, 1, and 4 mg/L respectively.

### Genome sequencing and analysis

The DNA of all isolates was extracted using the MOBIO kit (Qiagen) after an overnight growth in Luria-Bertani (LB) broth. The Nextera XT kit (Illumina, San Diego, California, USA) was used to prepare libraries. Sequencing was performed on a MiniSeq 2 × 150 cycles (Illumina technology). The SPAdes assembler was used to construct assemblies. Contigs <500 bp were removed. We performed multilocus sequence typing (MLST)^34^, identification of acquired resistance genes by using ResFinder 3.0^35^ and SerotypeFinder 1.1^36^.

The quality of the sequencing data was estimated using standard metrics. Mean N50 was of 266,195 bp and mean coverage was of 84. Quality sequencing data are summarized in Supplementary Table S4. Raw sequences of the 7 isolates were deposited in GenBank under BioProject number: PRJNA542787. Whole genome sequencing shows that *E. coli* was of ST131 and serotype O16 and had both *bla*_KPC-2_ and *bla*_TEM-1_, *E. cloacae* had the *bla*_KPC-3,_ *bla*_OXA-9_ and *bla*_TEM-1_ and the 5 *K. pneumoniae* were respectively of ST-258, ST-11, ST-101, ST-307 and ST-512 and produced either *bla*_KPC-2_ or *bla*_KPC-3_ (Supplementary Table S4).

### Determination of mutant frequencies

Mid-log cultures of either *E. coli* RD29 or *E. cloacae* RD26 in MH broth was diluted to 10^−4^ and cultured overnight with and without ACD. We evaluated seven ACD used in pediatric hematologic malignancies at concentrations close to the plasmatic concentrations^33^. The pyrimidine antagonist cytarabine was used at 0.5 mg/L^37^. The two antimetabolites azacitidine and mercaptopurine were used at 0.5 and 30 mg/L, respectively^38,39^. The two alkylating drugs cyclophosphamide and dacarbazine were used at 2.5 and 10 mg/L^40–42^. The topoisomerase inhibitor daunorubicin was used at 50 mg/L^37^. The anthracenedione mitoxantrone was used at 5 mg/L^43^.

We evaluated the basal mutation rate of the tested bacteria by plating appropriate dilutions of overnight cultures on MHA and MHA containing rifampicin (RMP) at 50 mg/L. The frequency of emergence of antibiotic-resistant mutants was calculated^20,21^ by plating overnight cultures on MHA and MHA containing 4 MIC of CZA (4:1 ratio) or meropenem (20 mg/l). We also evaluated the frequency of emergence of *E. cloacae* resistant to the combination CZA (4 MIC) – meropenem (MEM) 20 mg/l. This concentration of meropenem is compatible with the serum concentration obtained by continuous infusion of meropenem and above the MIC of the isolates^44^. All tests were done four times independently. In order to confirm and extend our results, five KPC-producing clinical isolates of *K. pneumoniae* of different ST were submitted to azacitidine on five independent experiments. The frequency of resistant mutants was then calculated as the number of resistant colonies divided by the number of plated cells. The results are expressed as the ratios of the mutant frequency with ACD on mutant frequency without ACD.

### MIC determination

MICs against CZA, MEM and MEM in combination with CZA were assessed with the E-test® method (bioMérieux, France) according the manufacturer’s recommendations. To determine the MIC of MEM combined to CZA, one CZA strip was placed on MHA for 30 min, then removed, and a MEM strip was placed on top of the gradient of the CZA^45^. The value at which the inhibition zone intersected the scale on the MEM E-test strip was considered as the MIC of MEM combined to CZA.

### Identification of mutations involved in the resistance to CZA

To identify the mutations leading to the resistance to CZA, *bla*_KPC_ of nine randomly selected mutants was PCR amplified using a high-fidelity polymerase (*Pfu* DNA polymerase) and sequenced with the primers KPC-F (5′ TCACTGTATCGCCGTCTAGTTCT-3′) and KPC-R (5′-ATCCCTCGAGCGCGAGTCTA-3′). (We did not search for mutations in the other *bla* genes that were present, nor in PBP’s (likely PBP3) that may also contribute to our results).

### Construction of plasmids expressing bla_KPC-3_ and derivatives

pBR322 plasmid was used as template for PCR amplification of the whole plasmid except *bla*_TEM_ using specific primers that also contained overlapping regions of 5′ and 3′ regions of *bla*_KPC_ (for subsequent Gibson Assembly) of RD26 isolate. *bla*_KPC_ was PCR amplified from the DNA extracts of RD26 and some of its CZA-resistant derivatives (RD26-2, -3, -5, -6, -7, -8, and -9) with specific primers that also contained overlapping regions of 5′ and 3′ regions of pBR322 plasmid. PCR products were purified with a 1-h digestion with *Dpn*I enzyme to remove the matrix DNA and by a gel purification (QIAquick Gel Extraction Kit, Qiagen). Resulting DNA was quantified using Qubit™ dsDNA HS Assay Kit (Thermofisher). Gibson reaction was carried out using a molar ratio of 1:1 backbone: insert in a total of 20 µl reaction mix and incubated at 50 °C for 1 hour.

The total volume of Gibson reaction was dialysed against distilled water for 30 min. One µl was then electroporated in 15 µl of One Shot™ TOP10 Electrocomp™ *E. coli* (Invitrogen) and then incubated in 500 µl of LB broth for 1 hour at 37 °C with shaking at 250 rpm. The transformants were plated on LB agar plates containing 10 mg/l tetracycline and further incubated overnight at 37 °C. Transformants obtained were PCR amplified using a high-fidelity *Pfu* DNA polymerase and sequenced with the primers pbr322-KPC-F GTCTGACAGTTACTGCCCGTTGA and pbr322-KPC-R AGGAAGAGTATGTCACTGTATCGCC.

### Quantitative reverse transcription-PCR (RT-PCR)

RNA from a Mueller Hinton broth culture was extracted with the Direct-zol RNA kit followed by a DNAse treatment (zymoresearch, Irvine, USA) as specified by the manufacturer.

Transcriptome analysis was performed using 10 ng of total RNA on 3 genes: *bla*_KPC_, *din*B a previously described housekeeping gene used for normalization^46^ and *tet*A, a pBR322 plasmidic gene coding for tetracyclin resistance also used for normalization. We used the following primers: KPC (F-CGCGGAACCATTCGCTAAAC, R-AGCCCTTGAATGAGCTGCAC), dinB (F-GCGCGATATCCCTATTGCTA, R-AGGCTTCTTTGTAGGCGTCA)^47^ and tetA (F- ATGCAGGAGTCGCATAAGGGAG, R- TCGCCGAAAATGACCCAGAG). The KAPA SYBR® FAST One-Step kit was used for PCR. Reverse transcription and amplification were performed with an LC480 Light Cycler (Roche®) in one step with the following cycling parameters: 30 min at 42 °C for reverse transcription, 3 minutes at 95 °C for reverse transcriptase inactivation and Taq activation followed by 45 cycles of 10 seconds at 95 °C, 20 seconds at 64 °C and 1 second at 72 °C. Assays were performed in triplicates. The cycle threshold (Ct) was automatically determined by using the Second Derivative Maximum Method included in the LC480 software. For each isolate, a mean Ct was calculated using the 3 replicates. The fold change in gene expression comparing mutants and pBR322-RD26 was calculated by using the 2^−ΔΔCt^ method^48^.

### Statistical analysis

The mutants frequencies were analyzed with the Fluctuation AnaLysis CalculatOR (FALCOR)^49^ program. We used the Ma-Sandri-Sarkar Maximum Likelihood Estimator (MSS-MLE) method; validated in low replication models^50^; followed by a Student’s *t*-test to conduct the statistical analysis. The chosen significance threshold was 0.05 for all tests.

### Accession numbers

Whole genome sequencing data were submitted to NCBI under SRA accession: PRJNA542787. *bla*_KPC_ genes were submitted to GenBank under accession numbers: RD26-3 MN091856; RD26-6 MN091857; RD26-7 MN091858; RD26-9 MN091859.

## Supplementary information

Supplementary Tables 1 to 5.

## Data Availability

All data generated or analysed during this study are included in this published article (and its Supplementary Information Files).
