# Supplementary material for: Impact of anticancer chemotherapy on the extension of beta-lactamase spectrum: an example with KPC-type carbapenemase activity towards ceftazidime-avibactam
Source: Sci Rep. 2020 Jan 17;10:589. doi: 10.1038/s41598-020-57505-w (PMC6969056; doi:10.1038/s41598-020-57505-w)
Supplement: Supplementary file 1 — Supplementary Tables 1 to 5. [file 41598_2020_57505_MOESM1_ESM.pdf]

Impact of anticancer chemotherapy on the extension of beta-lactamase spectrum: an example with KPC-type carbapenemase activity towards ceftazidime-avibactam

Authors: Claire Amaris Hobson[1], Stéphane Bonacorsi[1,2], Didier Hocquet[3], André Baruchel[4], Mony Fahd[4], Thomas Storme[5], Raksamy Tang[5], Catherine Doit[1,2], Olivier Tenaillon[1], André Birgy[1,2*].

[1]Université de Paris, IAME, INSERM, F-75018 Paris, France

[2]AP-HP, Hôpital Robert Debré, Service de Microbiologie, F-75019 Paris, France

[3] Hygiène Hospitalière, UMR CNRS 6249, Université de Bourgogne Franche-Comté, Besançon, France

[4] Service d'Immuno-Hématologie Pédiatrique, Hôpital Robert Debré, AP-HP, Paris, France

[5] Pharmacie Hospitalière, Hôpital Robert Debré, AP-HP, Paris, France

*__Corresponding author__: André Birgy (AB), PD, PhD, Hôpital Robert-Debré (APHP), 48 Boulevard Sérurier F75019 Paris, France (andre.birgy@aphp.fr). Phone +33 140034060

Supplementary Table S1:

Rates, average and standard deviation (sd) of resistant mutants of 2 strains of KPC-producing *Enterobacteriaceae* (RD26 and RD29) selected on 4 different antibiotic media (Ceftazidime/avibactam (CZA), Rifampicin (RMP), Meropenem (Mero) and the association Ceftazidime/avibactam and Meropenem (CZA-Mero)) after a culture with or without chemotherapy.

		**Resistance mutational rates to different antibiotics after exposure to cytotoxic molecules or non exposure (control)**															
**Isolate**	**Selective plate medium**	**Cytarabine**		**Mercaptopurine**		**Azacitidine**		**Daunorubicin**		**Dacarbazine**		**Cyclophosphamide**		**Mitoxantrone**		**Control**	
		Resistant mutational rates	average +/- sd (p-value)	Resistant mutational rates	average +/- sd (p-value)	Resistant mutational rates	average +/- sd (p-value)	Resistant mutational rates	average +/- sd (p-value)	Resistant mutational rates	average +/- sd (p-value)	Resistant mutational rates	average +/- sd (p-value)	Resistant mutational rates	average +/- sd (p-value)	Resistant mutational rates	average +/- sd
E. coli **KPC-2 (RD29)**	CZA 2mg/L	1,0E-06<br>1.7E-08<br>3.7E-08<br>1.4E-08	2.7E-07 +/- 4,9E-07 (0.15)	<1E-10<br>3.1E-08<br>1.0E-10<br>1.0E-10	7.7E-09 +/- 1.5E-08 (0.94)	<1E-10<br>1.9E-08<br>1.0E-10<br>1.0E-10	4.7E-09 +/- 9.2E-09 (0.87)	1.5E-07<br>1.0E-10<br>1.0E-10<br>1.8E-07	8.3E-08 +/- 9.7E-08 (0.83)	3.4E-06<br>1.3E-06<br>6.1E-06<br>2.4E-06	3.3E-06 +/- 2.1E-06 **(0.002)**	<1E-10<br>1.6E-08<br>4.5E-09<br>8.6E-08	2.6E-08 +/- 4.0E-08 (0.71)	4.5E-08<br>3.0E-08<br>8.3E-08<br>2.1E-07	9.3E-08 +/- 8.2E-08 **(0.04)**	4.0E-10<br>1.2E-09<br>1.6E-09<br>3.8E-08<br>5.5E-09	9.4E-09 +/- 1.6E-08
	RMP 50mg/L	1.5E-08<br>4.1E-08<br>6.2E-09<br>1.9E-08	2.0E-08 +/- 1.5E-08 (0.78)	4.5E-08<br>6.1E-08<br>3.7E-08<br>2.3E-08	4.2E-08 +/- 1.6E-08 (0.35)	3.7E-06<br>1.9E-06<br>1.4E-06<br>2.4E-06	2.3E-06 +/- 1.0E-06 **(0.003)**	3.0E-07<br>4.0E-06<br>3.0E-06<br>1.6E-05	5.8E-06 +/- 6.9E-06 (0.06)	1.8E-05<br>1.7E-05<br>5.9E-04<br>6.7E-06	1.6E-04 +/- 2.9E-04 **(0.005)**	3.9E-08<br>2.6E-08<br>1.3E-08<br>7.1E-08	3.8E-08 +/- 2.5E-08 (0.31)	6.5E-08<br>1.0E-07<br>5.0E-08<br>8.1E-09	5.6E-08 +/- 3.8E-08 (0.33)	1.6E-08<br>6.0E-08<br>5.5E-08<br>1.1E-08<br>1.8E-08	3.2E-08 +/- 2.3E-08
E. cloacae **KPC-3 (RD26)**	CZA 16mg/L	3.2E-09<br>7.2E-09<br>9.8E-09<br>4.6E-09	6.2E-09 +/- 2.9E-09 (0.98)	2.1E-08<br>3.7E-08<br>1.0E-08<br>2.8E-08	2.4E-08 +/- 1.1E-08 (0.1)	3.7E-07<br>3.8E-07<br>1.1E-06<br>1.0E-06	7.2E-07 +/- 4.0E-07 **(0.0006)**	1.3E-08<br>2.8E-08<br>2.4E-07<br>4.0E-07	1.7E-07 +/- 1.8E-07 (0.42)	1.2E-07<br>7.0E-08<br>3.0E-08<br>1.7E-07	9.6E-08 +/- 5.9E-08 **(0.003)**	1.1E-08<br>3.9E-09<br>7.4E-09<br>8.2E-09	7.6E-09 +/- 2.9E-09 (0.84)	3.6E-08<br>7.1E-08<br>1.7E-08<br>1.9E-08	3.6E-08 +/- 2.5E-08 **(0.008)**	6.1E-09<br>5.9E-10<br>2.6E-10<br>1.2E-08<br>1.1E-09	4.1E-09 +/- 5.2E-09
	RMP 50mg/L	4.4E-08<br>6.9E-08<br>2.5E-08<br>3.2E-08	4.2E-08 +/- 1.9E-08 (0.98)	2.5E-07<br>3.7E-08<br>4.8E-08<br>5.0E-08	9.5E-08 +/- 1.0E-07 (0.82)	1.6E-06<br>4.0E-06<br>2.4E-06<br>4.1E-06	3.0E-06 +/- 1.2E-06 **(0.001)**	1.3E-07<br>2.5E-07<br>3.5E-06<br>1.4E-06	1.3E-06 +/- 1.5E-06 (0.33)	2.5E-06<br>3.6E-07<br>1.8E-07<br>3.1E-07	8.5E-07 +/- 1.1E-06 (0.11)	2.5E-07<br>4.7E-08<br>7.4E-08<br>2.0E-07	1.4E-07 +/- 9.8E-08 (0.28)	1.1E-07<br>1.5E-07<br>1.7E-07<br>5.8E-08	1.2E-07 +/- 4.8E-08 (0.30)	7.6E-08<br>1.1E-07<br>6.3E-08<br>7.4E-08<br>4.8E-08	7.3E-08 +/- 2.1E-08
	MEM 20mg/L	*<br>*	*<br>*	*<br>*	*<br>*	*<br>*	*<br>*	*<br>*	*<br>*	*<br>*	*<br>*	*<br>*	*<br>*	*<br>*	*<br>*	*<br>*	*<br>*
	CZA-MEM	<1E-10<br><1E-10	0<br>0	<1E-10<br><1E-10	0<br>0	<1E-10<br><1E-10	0<br>0	<1E-10<br><1E-10	0<br>0	<1E-10<br><1E-10	0<br>0	<1E-10<br><1E-10	0<br>0	<1E-10<br><1E-10	0<br>0	<1E-10<br><1E-10	0<br>0

*Resistant rates were not evaluable since confluent growths were observed at this concentration

Supplementary Table S2:

Fold increase of resistant mutants' rates, average and standard deviation (sd) of 2 strains of KPC-producing *Enterobacteriaceae* (RD26 and RD29) selected on 4 different antibiotic media (Ceftazidime/avibactam (CZA), Rifampicin (RMP), Meropenem (Mero) and the association Ceftazidime/avibactam and Meropenem (CZA-Mero)) after a culture with or without chemotherapy, compared to the control group.

Fold increased[1] of resistant mutant rates to different antibiotics after exposure to different cytotoxics															
Isolates	Selective Plate medium	Cytarabine		Mercaptopurine		Azacitidine		Daunorubicin		Dacarbazine		Cyclophosphamide		Mitoxantrone	
		fold increase	average +/- sd	fold increase	average +/- sd	fold increase	average +/- sd	fold increase	average +/- sd	fold increase	average +/- sd	fold increase	average +/- sd	fold increase	average +/- sd
***E. coli* KPC-2 (RD29)**	CZA 2mg/L	2490.0	632.4 +/- 1238.4	0.0	4.7 +/- 9.3	0.0	3.8+/-7.7	373.5	101.7+/-181.8	8479.5	3416.2+/- 3660.9	0.0	6.4+/-7.7	113.2	57.0+/-38.9
		14.5		18.7		15.4		0.0		1051.3		9.6		25.2	
		22.6		0.0		0.0		0.0		3700.7		0.1		50.8	
		2.6		0.0		0.0		33.3		433.3		15.7		38.8	
	RMP 50mg/L	41.5	10.8+/-20.4	2.8	2.1+/-1.1	230.1	105.2+/-97.3	18.7	309.1+/-389.8	1103.7	3134.2+/- 5107.0	2.5	2.1+/-1.5	4.0	1.8+/-1.6
		0.7		1.1		31.0		73.2		288.3		0.5		1.7	
		0.1		3.4		25.0		274.5		10775.0		1.2		0.9	
		1.0		1.3		134.7		870.0		370.0		3.9		0.4	
***E. cloacae* KPC-3 (RD26)**	CZA 16mg/L	0.5	13.6+/-16.6	3.5	43.0+/-66.9	60.5	1492.5+/- 1925.6	2.1	125.2+/-170.9	19.4	102.2+/-58.0	1.8	6.2+/-6.5	6.0	52.1+/-51.7
		12.2		142.0		650.0		107.6		118.8		14.8		119.8	
		37.5		0.8		4329.3		19.1		115.8		0.6		64.7	
		4.3		25.8		930.0		372.0		155.0		7.6		18.0	
	MEM 20mg/L	*		*		*		*		*		*		*	
		*		*		*		*		*		*		*	
	RMP 50mg/L	0.6	0.6+/-0.1	3.3	1.4+/-1.3	21.2	45.4+/-27.1	1.7	20.4+/-21.5	33.6	11.6+/-14.7	3.3	2.3+/-1.7	1.4	1.7+/-0.6
		0.6		0.6		37.4		4.0		3.3		0.7		1.4	
		0.4		0.7		38.7		46.9		2.9		1.0		2.6	
		0.7		1.0		84.2		28.9		6.4		4.2		1.2	

[1]Four experiments were performed for each antibiotics

*Resistant rates were not evaluable since confluent growths were observed at this concentration

Supplementary Table S3:

Rates, average and standard deviation (sd) of resistant mutants of 5 strains of KPC-producing *Enterobacteriaceae* selected on 2 different antibiotic media (Ceftazidime/avibactam (CZA), Rifampicin (RMP), after a culture with or without chemotherapy.

	Mutation rate on RMP medium				Mutation rate on CZA medium			
	with chemotherapy	average +/- sd (p-value)	without chemotherapy	average +/- sd (p-value)	with chemotherapy	average +/- sd (p-value)	without chemotherapy	average +/- sd (p-value)
	3.66E-07		2.62E-08		6.21E-08		3.28E-10	
	4.56E-07		1.32E-08		1.32E-07		2.63E-10	
RD27	5.05E-07	4.97E-07 +/- 1.47E-07 **(<0.01)**	5.26E-08	2.32E-08 +/- 1.79E-08	1.29E-07	1.74E-07 +/- 1.48E-07 **(<0.01)**	4.39E-10	3.61E-10 +/- 7.39E-11
	7.43E-07		1.72E-08		4.34E-07		4.31E-10	
	4.15E-07		6.90E-09		1.12E-07		3.45E-10	
	4.85E-07		1.36E-08		1.29E-07		2.71E-10	
	3.51E-07		1.01E-08		1.70E-07		2.53E-10	
DD34	4.31E-07	3.98E-07 +/- 6.87E-08 **(<0.01)**	4.74E-09	1.14E-08 +/- 4.50E-09	5.38E-07	2.15E-07 +/- 1.96E-07 **(<0.01)**	7.11E-09	2.96E-09 +/- 3.63E-09
	4.11E-07		1.69E-08		2.24E-07		6.76E-09	
	3.10E-07		1.19E-08		1.63E-08		3.97E-10	
	1.13E-05		7.96E-07		6.71E-06		3.12E-07	
	7.16E-06		5.98E-07		5.39E-06		1.86E-07	
DD36	6.79E-05	1.99E-05 +/- 2.70E-05 **(<0.01)**	5.70E-07	6.11E-07 +/- 1.07E-07	3.49E-06	4.68E-06 +/- 1.76E-06 **(<0.01)**	9.00E-08	1.99E-07 +/- 1.04E-07
	3.95E-06		5.27E-07		2.30E-06		3.01E-07	
	8.98E-06		5.62E-07		5.52E-06		1.07E-07	
	4.69E-07		1.63E-08		8.97E-08		2.72E-10	
	1.37E-06		4.03E-09		3.17E-08		4.03E-10	
DD37	3.40E-06	1.29E-06 +/- 1.24E-06 **(<0.01)**	1.20E-08	8.43E-09 +/- 5.50E-09	1.04E-07	5.47E-08 +/- 3.89E-08 **(<0.01)**	3.98E-10	3.51E-10 +/- 5.87E-11
	4.30E-07		3.75E-09		2.65E-08		3.75E-10	
	7.61E-07		6.13E-09		2.17E-08		3.07E-10	
	4.54E-07		8.38E-09		3.21E-08		2.79E-10	
	7.64E-07		1.60E-08		1.39E-08		2.67E-09	
DD38	1.57E-06	8.04E-07 +/- 4.44E-07 **(<0.01)**	3.36E-08	1.77E-08 +/- 1.05E-08	1.70E-08	1.81E-08 +/- 8.53E-09 **(<0.01)**	2.58E-10	7.55E-10 +/- 1.07E-09
	6.76E-07		8.72E-09		1.80E-08		2.91E-10	
	5.57E-07		2.17E-08		9.43E-09		2.71E-10	

Supplementary Table S4:

Genome sequencing and analysis: identification of the beta-lactamases genes, sequence types and quality of the sequencing data illustrated by the mean N50 (bp), mean coverage and number of contigs.

Isolates	Genome_size	Number of contigs	n50	Coverage	Species	mlst	beta-lactamases genes
**RD26**	5020989	75	302644	101	*E. cloacae*	-	blaKPC-3,blaOXA-9,blaTEM-1A
**RD29**	4980967	90	309405	84	*Escherichia coli*	ST-131	blaKPC-2,blaTEM-1B
**RD27**	5542544	88	248804	80	*Klebsiella pneumoniae*	ST-512	blaKPC-3,blaOXA-9,blaSHV-11,blaTEM-1A
**DD34**	5619986	120	233237	72	*Klebsiella pneumoniae*	ST-258	blaKPC-2,blaOXA-9,blaSHV-12,blaTEM-1A
**DD36**	5747097	153	186314	75	*Klebsiella pneumoniae*	ST-11	blaKPC-2,blaSHV-11,blaTEM-1B
**DD37**	5710690	148	342058	75	*Klebsiella pneumoniae*	ST-101	blaKPC-2,blaOXA-1,blaOXA-9,blaSHV-1,blaTEM-1A
**DD38**	5579702	96	236649	106	*Klebsiella pneumoniae*	ST-307	blaKPC-3,blaOXA-9,blaSHV-28,blaTEM-1A

Supplementary Table S5:

Relative quantification of bla_{KPC} expression using transcription analysis by RT-qPCR of recombinant strains (*E. coli* Top10 with pBR322 harboring either wild-type allele (KPC-3) or alleles conferring ceftazidime-avibactam resistance. *din*B a chromosomal housekeeping gene and *tet*A a pBR322 plasmid gene (coding for tetracyclin resistance) were used for normalization.

Isolates	Replicates	Ct* *bla*KPC	mean Ct* KPC	Ct* *din*B	mean Ct* *din*B	Ct* *tet*A	mean Ct* *tet*A	Ct* *bla*KPC - Ct* chromosomal internal control	Ct* *bla*KPC - Ct* plasmidic internal control	delta delta Ct* chromosomal control	delta delta Ct* plasmidic control	Fold change compared to RD26 (chromosomal control)	Fold change compared to RD26 (plasmidic control)
Top10-pBR322-RD26 (KPC-3)	1	16.74		29.9		19.8							
	2	16.84	16.77	29.47	29.4	19.82	19.8	-12.6	-3.04	-	-	-	-
	3	16.73		28.71		19.81							
Top10-pBR322-RD26-7	1	16.5		29.18		19.94							
	2	16.72	16.61	29.2	29.2	19.91	19.9	-12.6	-3.3	0.0	-0.3	1.0	1.2
	3	16.6		29.27		19.85							
Top10-pBR322-RD26-1	1	17		29.8		20.48							
	2	17.01	17,0	29.72	29.7	20.43	20.4	-12.7	-3.4	-0.1	-0.4	1.1	1.3
	3	16.95		29.61		20.33							
Top10-pBR322-RD26-3	1	16.68		29.62		20.11							
	2	16.77	16.76	29.82	29.7	20.04	20.0	-13.0	-3.3	-0.4	-0.2	1.3	1.2
	3	16.81		29.68		19.93							
Top10-pBR322-RD26-6	1	16.78		29.49		20.19							
	2	16.89	16.8	29.62	29.6	20.07	20.1	-12.7	-3.3	-0.1	-0.2	1.1	1.2
	3	16.85		29.55		20.07							
Top10-pBR322-RD26-8	1	16.59		29.58		19.87							
	2	16.6	16.6	29.67	29.6	19.98	19.9	-13.0	-3.3	-0.4	-0.3	1.3	1.2
	3	16.55		29.59		19.79							
Top10-pBR322-RD26-5	1	15.8		28.12		18.24							
	2	15.86	15.8	28.17	28.1	18.26	18.3	-12.2	-2.4	0.4	0.6	0.8	0.7
	3	15.88		27.94		18.31							
Top10-pBR322-RD26-9	1	16.7		29.91		20							
	2	16.72	16.7	29.68	29.7	19.98	20.0	-13.0	-3.3	-0.4	-0.2	1.3	1.2
	3	16.74		29.53		20.03							
negative control	1	39.57	-	38.62	-	32.87	-	-	-	-	-	-	-

* Ct Cycle threshold

*din*B chromosomal gene used as control

*tet*A plasmid gene used as control
